# *Plasmodium berghei* High-Throughput (PbHiT): a CRISPR-Cas9 System to Study Genes at Scale

**DOI:** 10.21769/BioProtoc.5572

**Published:** 2026-01-20

**Authors:** Thorey K. Jonsdottir, Martina S. Paoletta, Johan Henriksson, Ellen S.C. Bushell

**Affiliations:** 1 The Laboratory for Molecular Infection Medicine Sweden, Umeå University, Umeå, Sweden; 2 Department of Molecular Biology, Umeå University, Umeå, Sweden; 3 Instituto de Agrobiotecnología y Biología Molecular (IABIMO), INTA-CONICET, Hurlingham, Buenos Aires, Argentina; 4 IceLab, Umeå University, Naturvetarhuset, Universitetsvägen, 901 87 Umeå, Sweden; 5 Umeå Center for Microbial Research (UCMR), Umeå University, Umeå, Sweden; 6 SciLifeLab, Umeå University, Umeå, Sweden

**Keywords:** *Plasmodium berghei*, *Plasmodium*, CRISPR-Cas9, Gene modification, High-throughput, Transfection, Malaria, Apicomplexan

## Abstract

Genetic modification is essential for understanding parasite biology, yet it remains challenging in *Plasmodium.* This is partially due to the parasite’s low genetic tractability and reliance on homologous recombination, since the parasites lack the canonical non-homologous end-joining pathway. Existing approaches, such as the *Plasmo*GEM project, enable genome-wide knockouts but remain limited in coverage and flexibility. Here, we present the *Plasmodium berghei* high-throughput (PbHiT) system, a scalable CRISPR-Cas9 protocol for efficient genome editing in rodent malaria parasites. The PbHiT method uses a single cloning step to generate vectors in which a guide RNA (gRNA) is physically linked to short (100 bp) homology arms, enabling precise integration at the target locus upon transfection. The gRNA also serves as a unique barcode, allowing pooled vector transfections and identification of mutants by downstream gRNA sequencing. The PbHiT system reliably recapitulates known mutant growth phenotypes and supports both knockout and tagging strategies. This protocol provides a reproducible and scalable tool for genome editing in *P. berghei*, enabling both targeted functional studies and high-throughput genetic screens. Additionally, we provide an online resource covering the entire *P. berghei* protein-coding genome and describe a step-by-step pooled ligation approach for large-scale vector production.

Key features

• PbHiT provides a high-throughput CRISPR-Cas9 genome editing platform optimised for *Plasmodium berghei* experimental infections in rodents.

• This protocol enables efficient and reproducible generation of knockout and tagged parasite lines using short homology arms.

• This protocol is supported by a free online resource for *P. berghei* gene construct design and requires basic knowledge of cloning.

• Transfection of *Plasmodium berghei* requires experience in handling mice/rats, an ethical permit, and an animal facility.

**This protocol is used in:** Nucleic Acids Research (2025), DOI: 10.1093/nar/gkaf005

## Graphical overview


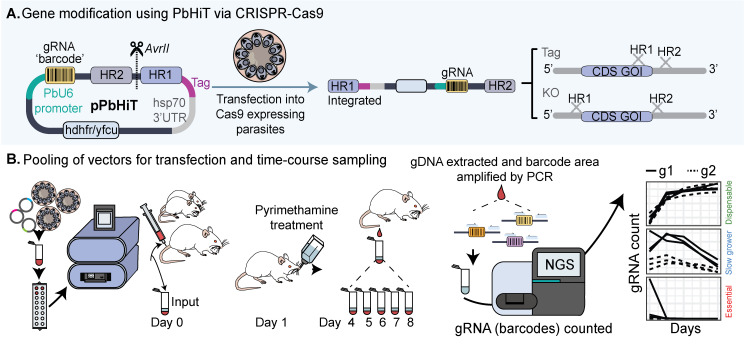


**Schematic overview of the *Plasmodium berghei* high-throughput (PbHiT) workflow.** (A) The pPbHiT vector (where p denotes plasmid) is designed with an h*dhfr/yfcu* drug selection cassette, facilitating both positive (h*dhfr*) and negative (y*fcu*) selection of the vector using pyrimethamine and 5-fluorocytosine, respectively. The synthetic fragment contains the gene-specific guide, homology region 2 (HR2) in the 3′ UTR of the target gene, and homology region 1 (HR1) either 100 bp upstream of the stop codon (tag) or start codon (knockout). The final vector is then linearised between HR2 and HR1 and integrated into the gene of interest in frame with the selected tag (Myc here), replacing the endogenous 3′ UTR with *hsp70* 3′UTR upon integration. (B) For scaling up, vectors are pooled together prior to transfection and injection into three individual mice. An input sample is taken on day 0, and then samples are taken daily once parasites are visible by blood smear (day 4 here), for 5 days. Genomic DNA (gDNA) is then extracted, and the barcode area is amplified by 2-step PCR, first by using generic primers (step 1) and then by using one generic primer and one index primer to label each sample (step 2). The PCR2 products are then pooled together (library) and analysed by next-generation sequencing (NGS).

## Background

Malaria is caused by *Plasmodium* parasites, which are injected into vertebrate hosts with the bite of a female *Anopheles* mosquito harbouring the parasite. It is estimated that 282 million cases were recorded in 2024, resulting in the death of 610 thousand people [1]. Reverse genetics has been critical to enable important studies of the parasite’s biology, to ultimately help inform future drug and vaccine intervention and treatment strategies [2]. *Plasmodium* gene editing, particularly in the human species *Plasmodium falciparum*, is challenging since the parasites lack the canonical non-homologous end-joining (NHEJ) pathway, have a very AT-rich genome (>80%), and have low transfection efficiency [3]. The rodent malaria species *Plasmodium berghei* is more genetically tractable, provides a model system to study gene essentiality in vivo, and provides efficient access to mosquito blood and liver stage infection, offering a more complete picture of those parasite genes important throughout the life cycle [4].

Several reverse genetic screens have been performed where a specific group of genes was targeted. This includes the knockout (KO) of all phosphatases and kinases in *P. berghei* [5,6], the systematic KO of the predicted exportome of *P. falciparum* [7], and the conditional mis-localisation of all non-secreted proteins on chromosome 3 in *P. falciparum* [8]. These types of gene-by-gene screens are immensely valuable but extremely time-consuming. More high-throughput KO screens have been completed at different stages of *P. berghei* (asexual blood stage, sexual commitment, fertilisation, and liver stage), with long-homology arm barcoded vectors from the *Plasmodium* Genetic Modification (*Plasmo*GEM) project covering more than half of the protein-coding genome. These barcoded vectors enabled the simultaneous transfection of 100× vectors into mice, where the relative growth of mutant parasites was estimated by tracking mutant barcodes over time using next-generation sequencing (NGS) [9–13]. However, the *Plasmo*GEM KO vectors could not be generated for the entire protein-coding genome due to technical limitations, and the scaling up of gene tagging vectors had limited success.

The adaptation of clustered regularly interspaced short palindromic repeats (CRISPR)-Cas9 gene editing in *Plasmodium* was done in 2014 [14–17]. In this system, the Cas9 endonuclease introduces a double-strand break at the desired target site, which is determined by the guide RNA (gRNA) sequence. Since *Plasmodium* does not have the classical NHEJ pathway, the repair is mediated by homologous recombination. Therefore, in addition to the presence of Cas9 and the gRNA, gene edits require a homology-directed repair (HDR) template [18,19]. The parasite also has a microhomology-mediated end-joining pathway, which has been previously used for CRISPR-Cas9 editing; however, this can only be used on genes that harbour repetitive sequences and therefore is not widely used [18,19]. CRISPR has been used successfully for large-scale KO screens in *Toxoplasma gondii*, another more genetically tractable apicomplexan parasite [20–24]. However, *T. gondii* has retained the NHEJ pathway, and therefore gene disruption vectors for large KO screens are much easier to generate and do not require the design or supply of an HDR template. Recently, a high-throughput tagging (HiT) system was designed for *T. gondii*, which relies on homologous recombination to introduce functionalised tags, where the vector contained a gRNA physically linked to the HDR template [25]. This opened the door to scalable CRISPR approaches in parasites that lack the NHEJ pathway.

Here, we present a detailed protocol for the *P. berghei* high-throughput (PbHiT) CRISPR-Cas9 system to target genes at a scale in *P. berghei*, inspired by the HiT approach for *T. gondii*. In our system, short homology arms (100 bp) are physically linked to the gRNA and integrated into the PbHiT vector using a single scalable cloning step. This system can be used for both gene tagging and gene KO, and the vector design can be done manually (as detailed here) or by using our online tool (https://pbhit-crispr-design.serve.scilifelab.se/search). Furthermore, the PbHiT vectors can be pooled together for transfection, where the gRNA serves as an NGS-readable barcode. PbHiT thereby offers the first high-throughput CRISPR system in *Plasmodium* parasites [26]. Finally, the sequencing data can be analysed and visualised using our scripts freely available at GitHub.

## Materials and reagents

**Biological materials**

1. XL10-Gold ultra-competent cells (Agilent Technologies, catalog number: 200315)

2. pPbU6-hdhfr/yfcu-HiT (Addgene, catalog number: 216421)

3. Other plasmid DNA (Genewiz-Azenta)

4. *Plasmodium berghei* ANKA Cas9 expressing parasite line (Bushell laboratory, Umeå University) [26]

5. Female BALB/c mice, at least 6 weeks of age (Charles River, Europe)

6. Female Wistar rats >150 g (Charles River, Europe)

**Reagents**

1. Oligonucleotides (Integrated DNA technologies) (see Table 1)

**Table 1.** Oligonucleotides used in this protocol

Name	Sequence (5′ → 3′)
PbU6prom_F	ACATATGCGCATACTTCGAGTTATAC
hsp70UTR_R	TATGTTCGTGGCATTCCACA
BC_pHiT_illumina_F	ACACTCTTTCCCTACACGACGCTCTTCCGATCTCGAATGCACTATTCATTTTATGGGG
BC_pHiT_illumina_R	TCGGCATTCCTGCTGAACCGCTCTTCCGATCTGACTCGGTGCCACTTTTTCA
PE 1.0 primer	AATGATACGGCGACCACCGAGATCTACACTCTTTCCCTACACGACGCTCTTCCGATC*T
Index primers	Sequences can be found in Bushell et al. [9]

*Note: All oligonucleotides except the first two were PAGE-purified.*

2. GeneJET Plasmid Miniprep kit (Thermo Fisher Scientific, catalog number: K0502)

3. BsmBI restriction enzyme (New England Biolabs, catalog number: R0580)

4. PstI restriction enzyme (New England Biolabs, catalog number: R0140L)

5. BbsI restriction enzyme (New England Biolabs, catalog number: R0539L)

6. AvrII restriction enzyme (New England Biolabs, catalog number: R0174L)

7. TAE buffer, 50× (VWR, catalog number: K915-4L; 1× TAE made up in distilled water and stored at room temperature)

8. Agarose (VWR, catalog number: 443666A; product format: made up to 0.7% or 1% in 1× TAE buffer)

9. 6× DNA loading dye (New England Biolabs, catalog number: B7024S)

10. T4 DNA ligase (New England Biolabs, catalog number: M0202L)

11. T4 DNA ligase reaction buffer (New England Biolabs, catalog number: B0202S)

12. Macherey-Nagel^TM^ NucleoSpin^TM^ Gel and PCR Clean-up (Macherey-Nagel, catalog number: 740609.250)

13. GoTaq G2 Green Master Mix (2×) (Promega, catalog number: M7822)

14. DreamTaq PCR Master Mix (2×) (Thermo Fisher Scientific, catalog number: K1081)

15. SYBR Safe DNA gel stain (Thermo Fisher Scientific, catalog number: S33102)

16. LB broth (Miller) (Sigma-Aldrich, catalog number: L3522; product format: made up in distilled water and autoclaved before use)

17. LB broth with agar (Miller) (Sigma-Aldrich, catalog number: L3147; product format: made up in water and autoclaved before use)

18. Ampicillin (Duchefa, catalog number: A0104, CAS: 69-52-3; product format: 100 mg/mL in water and stored at -20 °C)

19. Kanamycin (Duchefa, catalog number: K016, CAS: 25389-94-0; product format: 50 mg/mL in water and stored at -20 °C)

20. Ethanol (VWR, catalog number: 20821.310)

21. Sodium acetate (Sigma-Aldrich, catalog number: 567422)

22. Methanol (Sigma-Aldrich, catalog number: 34860)

23. Giemsa stain (Sigma-Aldrich, CAS: 51811-82-6; product format: made up to 10% in tap water and stored at room temperature)

24. Phosphate-buffered saline (PBS) (Sigma-Aldrich, Dulbecco, catalog number: D8537-500ML)

25. RPMI-1640 medium, HEPES (Fisher Scientific, Gibco, catalog number: 11594506)

26. Fetal bovine serum (FBS) (Gibco, catalog number: 10500-064)

27. Penicillin-streptomycin (Gibco, catalog number: 15140-122)

28. Sodium bicarbonate (Sigma-Aldrich, catalog number: S5761; product format: 1.2 M made up in distilled water)

29. Histodenz (Sigma-Aldrich, catalog number: D2158-100gr)

30. Heparin (Sigma-Aldrich, catalog number: H4784; product format: 50 mg/mL in Milli-Q water)

31. Pyrimethamine (MP Biomedicals, catalog number: 58-14-0; product format: 0.07 mg/mL in tap water, protect from light)

32. 5-fluorocytosine (Sigma-Aldrich, catalog number: F7129-5G; product format: 1 mg/mL in tap water, protect from light)

33. P3 Primary Cell 4D-Nucleofector X kit S (Lonza, catalog number: V4XP-3032)

34. DNeasy Blood and Tissue kit (Qiagen, catalog number: 69506)

35. Ketaminol (MSD Animal Health, catalog number: 51 15 19, 100 mg/mL)

36. Nerfasin/Xylazine (Dechra Veterinary Products, DIN 02444089, 20 mg/mL)

37. MinElute 96 UF PCR Purification kit (Qiagen, catalog number: 28051)

38. QIAGEN Plasmid Midi kit (Qiagen, catalog number: 12145)

39. Advantage 2 Polymerase mix (TaKaRa, catalog number: 639202)

40. Advantage UltraPure PCR deoxynucleotide mix (TaKaRa, catalog number: 639125)

41. Qubit 1× dsDNA HS Assay kit (Thermo Fisher Scientific, catalog number: Q33231)

42. UltraPure^TM^ DNase/RNase-free distilled water (Fisher Scientific, Invitrogen, catalog number: 11538646)

43. Parasite gas mixture: 1% oxygen, 3% carbon dioxide/nitrogen cylinder (Air Liquide Gas, catalog number: 228447-L)

44. Glycerol (Sigma-Aldrich, catalog number: G5516-100mL)

45. Alsever’s solution (MP Biomedicals, catalog number: 2801154)

46. Hydrochloric acid (Sigma-Aldrich, catalog number: 258148)

**Solutions**

1. Complete culture medium (see Recipes)

2. Histodenz solution (see Recipes)

3. Terminal anesthetic solution for mice (see Recipes)

4. Terminal anesthetic solution for rats (see Recipes)

5. LB broth with antibiotics (see Recipes)

6. LB agar plates with antibiotics (see Recipes)

7. Glycerol stock (see Recipes)

8. Parasite freezing solution (see Recipes)

**Recipes**

**1. Complete culture medium**

**Table:** 

Reagent	Final concentration	Quantity or volume
RPMI-1640 medium	72%	36 mL
FBS	25%	12.5 mL
Sodium bicarbonate	24 mM	1 mL of 1.2 M stock
Penicillin-streptomycin	1%	500 μL
Total		50 mL

Make fresh before each use.

**2. Histodenz solution**

To make a Histodenz stock solution (27.6%), first prepare 100 mL of buffered solution and adjust the pH to 7.5, then add the Histodenz (see below). Autoclave the solution (20 min at 120 °C) and store at 4 °C for up to 4 months. Wrap the bottle in aluminium foil to protect it from light.

**Stock solution**

**Table:** 

Reagent	Final concentration	Quantity or volume
*Buffered solution*		
Tris-HCl	5 mM	60.6 mg
KCl	3 mM	22.4 mg
CaNa_2_ EDTA	0.3 mM	12.3 mg
MilliQ water	-	Up to 100 mL total volume
*Histodenz stock solution*		
Buffered solution	-	100 mL
Histodenz	27.6%	27.6 g

*Note: Adjust pH using hydrochloric acid (HCl).*

**Caution:** Be careful when handling HCl and follow relevant safety guidelines.

Then, prepare in a 15 mL tube a fresh working solution of Histodenz (15.2%) in PBS (see below), just before using it.

**Working solution**

**Table:** 

Reagent	Final concentration	Quantity or volume
Histodenz stock solution	15.2%	2.75 mL
1× PBS	45%	2.25 mL
Total		5 mL

**3. Terminal anesthetic solution for mice**

**Table:** 

Reagent	Final concentration	Quantity or volume
Ketamine	33% (33.3 mg/mL)	2 mL
Xylazine	16.7% (3.33 mg/mL)	1 mL
PBS	50%	3 mL

Make the solution in a 15 mL tube and store at room temperature. The recipe can easily be scaled up if needed. Use a 25 G needle (or bigger) and a 1 mL Luer syringe to draw the solutions through the rubber lid of the glass containers. Do not use past the expiration date of the stock solutions, and always consult with your laboratory animal veterinarian. Weigh mice and use a dose of 225–270 mg/kg of ketamine and 47–60 mg/kg of xylazine.

**4. Terminal anesthetic solution for rats**

**Table:** 

Reagent	Final concentration	Quantity or volume
Ketamine	66.7% (66.7 mg/mL)	2 mL
Xylazine	33.3% (6.67 mg/mL)	1 mL

Make up the solution in a 15 mL tube and store at room temperature. Use a 25 G needle (or bigger) and a 1 mL Luer syringe to draw the solutions through the rubber lid of the glass containers. Do not use past the expiration date of the stock solutions, and always consult with your laboratory animal veterinarian. Weigh rats and use a dose of 225–270 mg/kg of ketamine and 15–30 mg/kg of xylazine.

**5. LB broth with antibiotics**

Dilute ampicillin or kanamycin stock 1:1,000 in LB broth before use.

**6. LB agar plates with antibiotics**

Melt the LB agar completely by placing the bottle in a water bath (42 °C) or in a microwave. Once melted, wait for the agar to cool down to ~50 °C before adding ampicillin or kanamycin (1:1,000 dilution), mix gently, and pour onto plates (enough to cover the surface area). Let agar settle at room temperature; once solid, stack plates together and store in the fridge (up to approximately 1 month).

**7. Glycerol stock**

Make up a 50% glycerol stock solution in Milli-Q water and autoclave. Store at room temperature. Mix 1:1 bacteria culture and glycerol stock solution to make up a 25% glycerol stock. Store bacteria glycerol stock at -80 °C.

**8. Parasite freezing solution**

Mix in a 50 mL tube 5 mL of 100% glycerol and 45 mL of Alsever’s solution. Mix well and filter-sterilise using a 0.2 μm filter. Store 10 mL aliquots at -20 °C for long-term storage or at 4 °C for short-term storage.

**Laboratory supplies**

1. 1.5 mL safe-lock tubes (Fishes Scientific, Eppendorf, catalog number: 15625367)

2. Screw cap tube, 15 mL (Sarstedt, catalog number: 62.554.502)

3. Screw cap tube, 50 mL (Sarstedt, catalog number: 62.547.254)

4. Falcon polypropylene round-bottom test tubes (loose cap) (Merck, Corning, catalog number: CLS352006)

5. No. 23 scalpel blade (Scientific Laboratory Supplies, Swann-Morton, catalog number: INS4688)

6. Microscope slides (Thermo Scientific, Epredia, catalog number: 16247920)

7. Narrow mouth Erlenmeyer flask, reusable (Merck, Corning, catalog number: CLS4985P500)

8. BD PlastiPak^TM^ Luer slip syringe without needle (Thermo Fisher, catalog number: 15489199)

9. 25 G needles (Fisher Scientific, BD Microlance, catalog number: 10442204)

10. Omnican 20 U insulin injection syringe (Fisher Scientific, B Braun, catalog number: 15188928)

11. Sterile blood lancets (Vitrex Medical, catalog number: 357213)

12. Benchtop cooler (Thermo Scientific, catalog number: 10777232)

13. Polypropylene micro tube rack, pack of 5 (Thermo Fisher, catalog number: 11728084)

14. Delrin full-size test tube racks, 24 mm × 30 mm (Thermo Fisher, catalog number: 10257963)

15. 1.8 mL cryotubes (Thermo Scientific, catalog number: 368632)

16. 5 mL serological pipettes (VWR, catalog number: 612-3702)

17. 10 mL serological pipettes (VWR, catalog number: 612-3700)

18. 25 mL serological pipettes (VWR, catalog number: 612-3698)

19. PCR tubes (VWR, catalog number: 732-0545)

20. 10 μL tips (VWR, catalog number: 613-6463)

21. Tips 200 μL sterile filtered tips (VWR, catalog number: 732-3397)

22. 1,000 μL tips (VWR, catalog number: 613-6471)

23. 90 mm plates (Thermo Scientific, catalog number: 101VR20)

24. Spreader, T-shaped (VWR, catalog number: 612-2651)

25. Reagent reservoirs (VWR, catalog number: 613-1181)

26. 96-well deep-well plate (VWR, catalog number: 732-2893)

27. Pasteur pipette (VWR, catalog number: 612-1681)

28. PCR microplate (Axygen, catalog number: PCR-96-LP-AB-C)

29. Microplate sealing film, rayon (Fisher Scientific, Axygen, catalog number: 11326254)

30. Microplate sealing film, aluminium (Fisher Scientific, Axygen, catalog number: 12577947)

31. Millex^TM^-GS filter unit (sterile) (Merck, Millipore, catalog number: SLGS033)

## Equipment

1. Eppendorf Research plus, 4-pack (Eppendorf, catalog number: 3123000950)

2. Pipetboy acu 2 (VWR, catalog number: 612-2964)

3. Beckman Coulter centrifuge (Beckman Coulter, model: Allegra V-15R, catalog number: C63126)

4. Pico^TM^ 21 microcentrifuge (Thermo Fisher Scientific, catalog number: 75002553)

5. C1000 Touch^TM^ thermal cycler with 96-well fast reaction module (Bio-Rad, catalog number: 1851196)

6. Eppendorf ThermoMixer C (Merck, Eppendorf, catalog number: EP5382000015)

7. Eppendorf ThermoTop (Merck, Eppendorf, catalog number: EP5308000003)

8. IKA KS 4000 i incubator shaker (Fisher Scientific, IKA, catalog number: 12944035)

9. Benchtop orbital shaker (Eppendorf, New Brunswick, model: Innova^®^ 40, catalog number: M1299-0092)

10. DUALED blue/white transilluminator (Bioneer, catalog number: A-6020)

11. ChemiDoc imaging system (Bio-Rad, catalog number: 12003153)

12. Amaxa 4D-Nucleofector^TM^ core unit (Lonza, catalog number: AAF-1002B)

13. D-Nucleofector^®^ X unit (Lonza, catalog number: AAF-1003X)

14. Counting chamber with double net ruling and V-slash (Fisher Scientific, Marienfeld Superior, catalog number: 15110535)

15. UVP PCR3 HEPA workstation (analytik jena, catalog number: 849-95-0600-02)

16. QIAvac connecting system (Qiagen, catalog number: 19419)

17. QIAvac 96 (Qiagen, catalog number: 19504)

18. Mice incubator (Vet-Tech, VTS Serial No. 1458, Code No. HEO11)

19. NGS sequencing platform (e.g., Illumina MiSeq, NextSeq, NovaSeq)

20. Nanodrop (IMPLEN, catalog number: T81946)

21. Lab bench (DanLaf VFRS 1206, Scanbur, catalog number: 5522190A) and suction system N840 Laboport (KNF, catalog number: 5220 19 0612, Hose connector, catalog number: 317278, key for hose connector, catalog number: 316279, spare parts kit, catalog number: N 840 G 317436)

22. PCR roller (Merck, catalog number: AXYPCRSPROLLER)

23. 8-channel multichannel pipette (Eppendorf, catalog number: 3125000036)

## Software and datasets

1. EuPaGDT tool: http://grna.ctegd.uga.edu/ [27] EuPaGDT: a web tool tailored to design CRISPR guide RNAs for eukaryotic pathogens, free

2. PlasmoDB: https://plasmodb.org/plasmo/app [28] VEuPathDB: the eukaryotic pathogen, vector, and host Bioinformatics Resource Center, free

3. Benchling (Biology software): 2024, https://www.benchling.com/, free

4. NEBio Calculator (New England Biolabs, v1.15 and v1.16): https://nebiocalculator.neb.com/, free

5. Image Lab Software, Bio-Rad (Version 6.1) (SOFT-LIT-170-9690-ILSPC-V-6-1), free

6. R: https://www.r-project.org/ [29], free

7. RStudio: http://www.posit.co/ [30], free

8. PbHiT data analysis and visualisation pipeline: https://github.com/henriksson-lab/pbhit_analyzer, free

9. R package minpack.lm v.1.2–4: https://CRAN.R-project.org/package=minpack.lm [31] *minpack.lm: R Interface to the Levenberg-Marquardt Nonlinear Least-Squares Algorithm Found in MINPACK, Plus Support for Bounds.* R package

10. RShiny: https://cran.r-project.org/web/packages/shiny/index.html [32] *shiny: Web Application Framework for R.* R package version 1.9.0.

11. ggplot2: https://ggplot2.tidyverse.org [33] ggplot2: Elegant Graphics for Data Analysis.

12. plotly: https://plotly-r.com [34] Interactive Web-Based Data Visualisation with R, plotly, and shiny.

13. PbHiT online design tool: https://pbhit-crispr-design.serve.scilifelab.se/search [26].

## Procedure

Sections A–F describe the standard PbHiT workflow for generating and validating individual gene targeting vectors and *P. berghei* mutants. This part of the protocol is suitable for users working with a small number of targets and wishing to generate vectors and mutants for conventional single-gene targeting studies. Sections G–J provide an adaptation of this system for pooled large-scale mutagenesis using gRNA sequencing (PbHiT-Seq) to phenotype mutants, designed for projects targeting tens to hundreds of genes simultaneously. This latter part of the protocol generates pools of vectors and mutants for analysis in a pooled format, where vectors and mutants from within the pool can be identified by NGS. Section G can be adapted to generate individual gene targeting vectors by a pooled-ligation protocol, where arrayed individual vectors can be identified by Sanger sequencing. Users might consider using the pooled-ligation approach to make >12 individual vectors.

**A. Designing the gRNA-HDR template**

1. Choose the gRNA sequence for your gene of interest using the EuPaGDT tool.

a. Find the genomic sequence of your specific gene, including 500 bp of the 5′ and 3′ untranslated region (UTR) using the *Plasmo*DB database.

b. Select the best guide(s) based on the ranking of the gRNA and the proximity to the site of editing (important for tagging constructs only) as determined by the tool. For KO constructs, the gRNA can be localised anywhere within the coding DNA sequence (CDS). For tagging constructs, choose an area in the 3′ UTR as close to the stop codon as possible (preferably within 50–100 bp).

**Figure 1.**
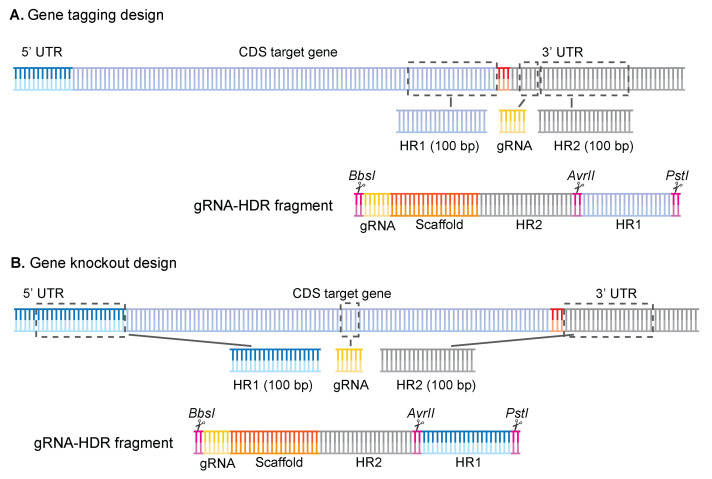
Gene tagging and gene knockout designs for the gRNA-HDR fragment. Overview of how the homology regions and gRNAs were selected for both (A) gene tagging and (B) gene knockout approaches. For gene tagging, HR1 is chosen before the stop codon, the gRNA is chosen in the 3′ UTR as close to the stop codon as possible, followed by the HR2 fragment. For gene knockout, homology regions are chosen before the start codon (HR1) or after the stop codon (HR2), and gRNA can be placed anywhere within the coding DNA sequence (CDS). The synthetic fragment is then ordered in the following order for both designs: BbsI/gRNA/gRNA-scaffold/HR2/AvrII/HR1/PstI. Figure adapted from Jonsdottir et al. [26].

2. Design your homology regions (Figure 1).

a. For KO designs, select a 100 bp area just before the start codon and use it as homology region 1 (HR1), and then select a 100 bp area immediately after the stop codon and use it as homology region 2 (HR2).

b. For tagging designs, select a 100 bp area just before the stop codon and use it as HR1, and then select a 100 bp area 6 bp downstream of the protospacer adjacent motif (PAM) when the gRNA is on the forward strand, or immediately after the gRNA sequence if the gRNA is on the reverse strand.

3. Assemble your gRNA-HDR fragment in sequence software and check if everything is correct before ordering.

a. Double-check that your HR regions do not contain cut sites for the following restriction enzymes: BbsI, PstI, or AvrII. If they do, you must introduce synonymous codon substitutions in these regions to remove the cut site.

**Critical:** It is crucial that these restriction sites are not present in your template, as it will result in incorrect ligation, which can be missed if using the pooled format (section G).

b. Now, combine the sequences you selected in the above steps to generate the gRNA-HDR template using software such as Benchling in the following order (see Figure 1): BbsI/gRNA/gRNA-Scaffold/HR2/AvrII/HR1/PstI or GAAGACggTATT/(N20)/GTTTTAGAGCTAGAAATAGCAAGTTAAAATAAGGCTAGTCCGTTATCAACTTGAAAAAGTGGCACCGAGTCGGTGC/HR2/CCTAGG/HR1/CTGCAG. Make sure that you have the gRNA in the correct orientation.

c. Order the sequence as a synthetic gene fragment from Genewiz-Azenta or a similar service in a plasmid. Here, the synthetic fragment was ordered in a standard pUC-GW-Kan cloning vector from Genewiz-Azenta, containing the kanamycin (kan) resistance cassette.

*Note: Synthetic fragment designs can be found for all* P. berghei *protein-coding genes using our online tool:* *https://pbhit-crispr-design.serve.scilifelab.se/search* . *If using the tool, be careful to check the fragment (restriction sites, gRNA orientation, and the location of homology regions) prior to ordering.*

**B. Prepare the gRNA-HDR template for cloning**

1. Prepare the pUC-GW-Kan vector for cloning.

a. Spin down (quick spin) the tubes containing the lyophilised synthetic fragment cloned into the pUC-GW-Kan vector and add water to a final concentration of 0.1 μg/μL. Vortex and let sit on ice for 10 min.

b. Transfer 1 μL of the plasmid into a 1.5 mL tube together with 99 μL of water to dilute the plasmid.

c. Transform 1 μL of the diluted plasmid into chemically competent *E. coli* by incubating on ice for 30 min, before giving the cells a heat shock for 30 s on a heat block at 42 °C, and putting them back on ice for 2 min.

*Note: When transforming the pUC-GW-Kan vector, in-house competent cells that might be less efficient than commercial cells can be used since only one colony is needed.*

d. Resuspend cells in 500 μL of warm LB broth, transfer to a loose cap tube, and let recover for up to 1 h at 37 °C whilst shaking at 225 rpm.

e. Spread 100 μL of the media with your cells into an LB agar plate containing kanamycin and incubate overnight at 37 °C.

**Pause point:** After the overnight incubation, the agar plate can be stored at 4 °C and colonies picked another day. We recommend not waiting longer than two weeks, as there is a risk of contamination whilst storing the plate.

f. Next day, pick a single colony and place it in 4 mL of LB containing kanamycin and incubate for around 16 h at 37 °C whilst shaking at 225 rpm.

g. The next day, spin down the bacterial culture and purify plasmid DNA using a miniprep kit according to the kit instructions. Optional: Make glycerol stocks from the transformed bacteria for future use by resuspending 50% bacteria culture with 50% glycerol and freeze at -80 °C.

**Pause point:** The protocol can be paused here, and the plasmid can be stored at -20 °C until used.

2. Digest the pUC-GW-Kan vector to release the gRNA-HDR fragment.

a. Set up a restriction digest in a 1.5 mL tube by adding 1 μg of pUC-GW-Kan plasmid DNA, 0.5 μL of BbsI, 0.5 μL of PstI, 5 μL of SmartCut buffer, and water up to 50 μL.

b. Incubate the reaction overnight on a heat block at 37 °C.

c. The next day, add 10 μL of 6× DNA loading dye to the digest and run all the digest on a 1% agarose gel.

d. Visualise the bands on the agarose gel using a transilluminator without exposing the DNA to UV light.

e. Cut out the gRNA-HDR fragment (320 bp) using a scalpel blade and transfer the gel piece into a 1.5 mL tube. Purify DNA according to the NucleoSpin Gel and PCR Clean-up kit protocol. Elute in 15 μL of water for higher concentration.

**Caution:** Handle the scalpel blade with care.

f. Measure the DNA concentration using Nanodrop.

**Pause point:** The protocol can be paused here, and the plasmid can be stored at -20 °C until used.

**C. Prepare the PbHiT vector and the ligation of the gRNA-HDR template**

**Figure 2.**
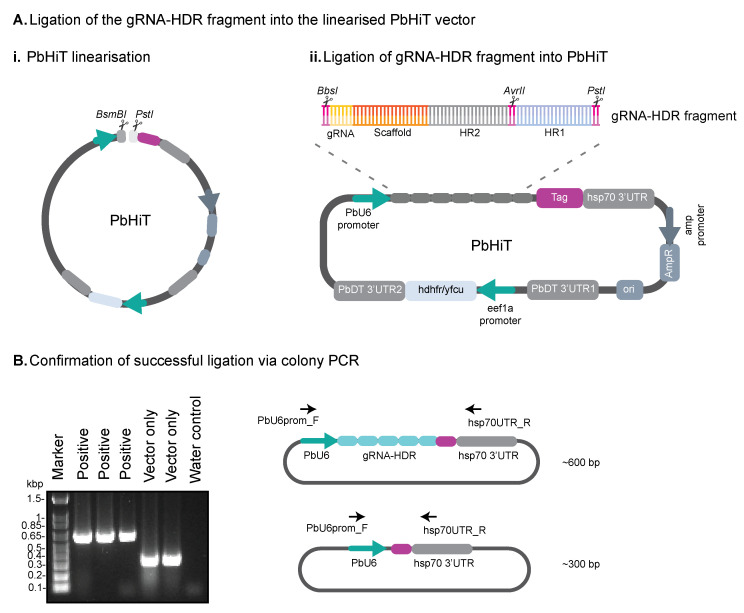
Confirming successful ligation of the gRNA-HDR fragment into the PbHiT vector. (A) (i) The PbHiT vector is linearised using BsmBI and PstI restriction enzymes and then (ii) the gRNA-HDR fragment is ligated into the PbHiT vector. (B) Successful ligation is confirmed using colony PCR, where the vector only produces a band around 300 bp, and successful ligation shows a band around 600 bp.

1. Digest the PbHiT vector with BsmBI (see Figure 2Ai).

a. In a 1.5 mL tube, add 4 μg of PbHiT plasmid DNA, 2 μL of BsmBI, 5 μL of SmartCut buffer, and water up to 50 μL.

b. Incubate the reaction on a heat block at 55 °C overnight.

c. Next day, confirm that the vector has been linearised by running 1 μL of the digest on a 0.7% agarose gel. To do this, add 1 μL of plasmid DNA, 1 μL of 6× DNA loading dye, and 4 μL of water on parafilm and load on the gel.

d. Purify the remaining linearised vector directly using the NucleoSpin Gel and PCR Clean-up kit. We recommend eluting the plasmid in 30 μL of warm (56 °C) elution buffer or water. Repeat the elution step by passing the elution through the column again to increase the yield.

e. Measure the DNA concentration using Nanodrop.

**Pause point:** The protocol can be paused here, and the plasmid can be stored at -20 °C until used.

2. Digest the PbHiT vector subsequently with PstI (see Figure 2Ai).

a. In a 1.5 mL tube, add all the PbHiT plasmid DNA prepared in step C1. Then, adjust PstI enzyme volume according to plasmid concentration, add 5 μL of SmartCut buffer, and water up to 50 μL. Then incubate the reaction on a heat block at 37 °C overnight.

b. Purify the linearised vector directly using the NucleoSpin Gel and PCR Clean-up kit. We recommend eluting the plasmid in 30 μL of warm (56 °C) elution buffer or water. Repeat the elution step by passing the elution through the column again to increase the yield.

c. Measure the DNA concentration using Nanodrop.

**Pause point:** The protocol can be paused here, and the plasmid can be stored at -20 °C until used.

3. Ligate the gRNA-HDR fragment (prepared in step B2) into the linearised PbHiT vector (see Figure 2Aii).

a. Use the NEBio Calculator to calculate how much insert you need for a ligation using 50 ng of vector and a 3:1 ratio (insert:vector).

b. Set up a ligation reaction in a 1.5 mL tube by adding 50 ng of vector, x ng of insert (as calculated in step C3a), 1 μL of T4 DNA ligase reaction buffer, 1 μL of T4 DNA ligase, and water up to 10 μL.

c. Incubate the reaction overnight at 4 °C.

d. Next day, transform 2 μL of ligation reaction into chemically competent cells as described in step B1.

*Note: Leftover ligation reaction can be stored at -20 °C to use later if needed. Label the tube clearly.*

4. Confirm integration of gRNA-HDR into PbHiT vector using colony PCR and sequencing (see Figure 2B).

a. Set up a PCR master mix of 50 μL using 25 μL of 2× GoTaq polymerase mix in a PCR tube.

b. Add 1 μL of PbU6prom_F and 1 μL of hsp70UTR_R primers (Table 1).

c. Add water up to 50 μL and then transfer 12.5 μL of PCR mix into 4× PCR tubes.

d. Pick a single colony with a tip, lightly dab it on an LB agar plate (+ampicillin), and then swirl the same tip in one of the PCR tubes. Do the same for two other colonies and leave the last tube as a water-only control.

e. For colony PCRs, the initial denaturing step is set to 95 °C for 2 min, then 30× cycles of 95 °C for 15 s, 50 °C annealing temperature for 30 s, 62 °C extension temperature for 1 min, and the final extension at 62 °C for 5 min.

f. Run 5 μL of each PCR reaction on a 1% agarose gel to confirm integration of gRNA-HDR into PbHiT. A band should be visible around 600 bp if the ligation was successful.

**Pause point:** The agar plate can be stored at 4 °C, and positive colonies can be used later. We recommend not waiting longer than two weeks, as there is a risk of contamination whilst storing the plate.

g. Once ligation has been confirmed, grow positive colonies in LB broth + ampicillin overnight at 37 °C and 225 rpm, purify the plasmid DNA the next day using the Miniprep kit, and send for Sanger sequencing.

*Note: We recommend making a glycerol stock from the bacterial culture before purifying DNA.*

**Pause point:** The protocol can be paused here, and the plasmid can be stored at -20 °C until used.

5. Linearise the final PbHiT-gRNA-HDR vector with AvrII.

a. In a 1.5 mL tube, set up a digest reaction by adding 2 μg of the final vector, 1 μL of AvrII, 5 μL of SmartCut buffer, and water up to 50 μL.

b. Incubate the digest on a heat block at 37 °C overnight.

c. Next day, add 10 μL of DNA loading dye to the digest and run the whole digest on a 0.7% agarose gel.

d. Visualise the bands on the agarose gel using a transilluminator, cut out the linearised vector, and purify the DNA using the NucleoSpin Gel and PCR Clean-up kit as before, using a double elution step.

**Pause point:** The protocol can be paused here, and the DNA can be stored at -20 °C until used.

e. Precipitate the DNA in a 1.5 mL tube for transfection by adding 1/10 volume of sodium acetate and 2.5 volumes of 100% ice-cold ethanol. Then, incubate at -20 °C overnight.

f. The next day, centrifuge the tube at 16,000× *g* for 20 min at 4 °C. Remove the supernatant and add (do not resuspend) 500 μL of 70% ice-cold ethanol. Centrifuge again for 10 min and repeat the wash once more.

g. Dry the pellet at room temperature for around 5 min, or until the pellet is dry (be careful not to overdry the pellet).

h. Resuspend the pellet in 8 μL of Milli-Q water and run 1 μL on a 0.7% agarose gel to confirm the presence of the linearised plasmid prior to transfection.

**Pause point:** The protocol can be paused here, and DNA can be stored at 4 °C until used. If storing the DNA, run the DNA on a gel after storing to confirm the DNA is good for transfection.

**D. Infect mice with Cas9-expressing parasites and set up a schizont culture**

**Caution:** Handle mice, needles, and terminal anaesthesia using appropriate precautions.

1. Infect a Balb/c mouse with the *P. berghei* ANKA Cas9-expressing parasite line.

a. Thaw a frozen parasite stock of the *P. berghei* ANKA Cas9-expressing parasite line and bring it to room temperature.

*Note: Carry the cryovial of frozen stock in a benchtop cooler to prevent prolonged exposure to room temperature prior to injection.*

b. Inject 200 μL of the parasite stock intraperitoneally into a Balb/c mouse.

c. About 4 days post-infection, the mouse should have high enough parasitemia (3%–5%) to set up a parasite culture.

*Note: For single transfections, one mouse will yield enough schizonts to transfect 2–3 vectors (single transfection) as the aim is to have at least 2 μL of purified schizont pellet. For vector pools, we recommend first infecting one mouse, and then infecting a rat using the infected mouse’s blood, especially if working with larger vector pools (>25 vectors per transfection).*

2. Set up a schizont culture for transfection.

a. Prepare 50 mL of complete culture media in a flask.

b. When the mouse has reached ideal parasitemia, prepare terminal anaesthesia and inject intraperitoneally into the Balb/c mouse according to Diehl et al. [35] or any specific institutional guidelines for the care and use of laboratory animals.

c. Draw up 100 μL of heparin into a 1 mL syringe and prepare an empty 1.5 mL tube.

d. Collect the blood via heart puncture (should get around 1 mL) and immediately euthanise the mouse.

e. Add the blood into the flask with the prepared media and gas (3% CO_2_, 1% O_2_, and 96% N_2_).

f. Incubate the parasite cultures for 22 h at 37 °C whilst shaking at 80 rpm.

*Note: Appropriate training is necessary before handling animals.*

**E. Transfect the PbHiT-gRNA-HDR linearised vector into Cas9-expressing schizonts**

1. Use a 15.2% Histodenz/PBS gradient to purify late-stage schizonts from a 22 h culture.

a. In a 15 mL centrifuge tube, mix 2.75 mL of Histodenz stock and 2.25 mL of PBS (final concentration 15.2%).

b. Transfer the schizont culture to a 50 mL tube and centrifuge at 300× *g* for 14 min with acceleration/deceleration set to 1. All centrifugation steps in this section should be completed at room temperature.

c. Remove the supernatant but leave around 3 mL of media with the blood pellet.

d. Resuspend the blood pellet and the media using a Pasteur pipette and layer slowly on top of the Histodenz.

**Critical:** It is important to layer the schizont culture slowly on top of the Histodenz and to make sure it does not mix together.

e. Centrifuge at 300× *g* for 20 min with acceleration/deceleration set to 1.

**Caution:** Transfer tubes from the centrifuge to the tube rack with great care to avoid mixing the schizont layer with the media/Histodenz above and below.

f. Collect the brown layer in the middle, above the Histodenz, which contains the schizonts, and transfer to a new 15 mL Falcon tube.

g. Add RPMI (room temperature) to the collected schizonts (up to 15 mL) to wash away any Histodenz. Centrifuge at 450× *g* for 3 min with acceleration/deceleration set to 3.

h. Remove all the supernatant from the schizont pellet.

*Note: Start on steps E2a–b during the 20-min centrifugation in step E1e.*

2. Transfection of the plasmid into schizonts and injection into mice.

a. Turn on the heat box to 37 °C and place the mice inside.

b. Fill one insulin syringe per transfection with 100 μL of RPMI (room temperature). Make sure to label the syringes if doing more than one transfection to avoid confusion.

**Caution:** It is important to label syringes correctly; otherwise, you can end up with issues later.

c. Add the relevant amount of P3 buffer (supplemented) to the schizont pellet. You need at least 2 μL of pure schizont pellet per transfection. Higher amounts can also be used without adverse effects. If only doing one transfection, resuspend the pellet in 18 μL of P3 buffer. If doing two transfections, resuspend the pellet in 2 × 18 μL of P3 buffer, and so forth.

d. Transfer 20 μL of schizont pellet + P3 buffer to the tube that contains 7 μL of plasmid DNA. Resuspend and transfer 26 μL into one well in a 16× Nucleocuvette strip. Make sure you do not introduce any bubbles.

**Critical:** It is important not to have any bubbles, as this can result in an error during transfection.

e. Immediately place the cuvette strip in the Lonza machine using the cuvette setting, then choose P3 as your primary cell solution and programme FI 115. Then, start the experiment.

f. Immediately after electroporation, add the 100 μL of RPMI from the insulin syringe prepared in step E2b into the relevant well and then take up everything, making sure to avoid touching the plastic with the needle to prevent it from going blunt. Remove any air bubbles from the syringe before proceeding.

**Caution:** It is important not to touch the plastic with the needle, since this can make the tip blunt and cause issues during the intravenous injection.

g. Inject the schizont/plasmid/RPMI mixture into the lateral caudal vein of a mouse.

*Note: Aim to inject the schizonts into the mice relatively quickly and do not prepare more than 3–4× transfections at the same time.*

h. Provide pyrimethamine drinking water to the infected mouse the day after transfection. Wrap the drinking flask in aluminium foil, since pyrimethamine is light-sensitive.

i. Monitor parasitemia daily using smears from a tail bleed as described in step F1.

**F. Monitor parasitemia and confirm correct integration using PCR**

1. Monitor parasitemia using daily smears from a tail bleed.

a. Use a lancet to gently prick the end of the mouse tail, add a blood drop to a glass slide, and make a thin smear.

b. Fix the smear in 100% methanol for 30 s and then stain in 10% Giemsa stain for 10 min.

c. Add oil to the dry-stained smear and check parasitemia by using a light microscope and 100× lens.

d. Once parasitemia reaches ~3%, collect the blood into a tube via cardiac puncture as described in steps D2b–d.

*Note: Parasites are normally visible by blood smear on day four post-transfection using the PbHiT approach, but this will depend on the target gene and guide used.*

e. Use 20 μL of the blood for genomic DNA extraction. The remaining blood can be used to prepare frozen parasite stocks by mixing one part of blood with one part of freezing solution. Place immediately at -80 or -120 °C.

2. Genomic DNA extraction.

a. Resuspend the 20 μL of infected blood with 180 μL of PBS and then follow the Qiagen DNeasy Blood and Tissue kit protocol from step F1d.

b. Perform double elution in 100 μL of elution buffer.

**Pause point:** The protocol can be paused here, and gDNA can be stored at 4 or -20 °C.

**Figure 3.**
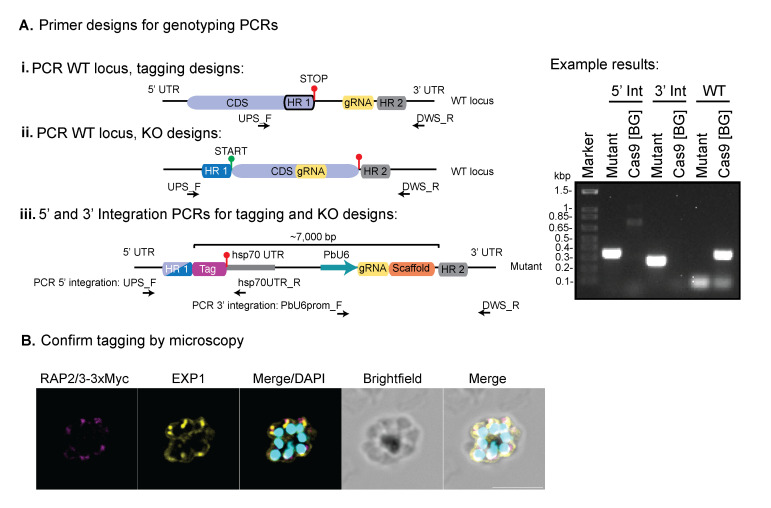
Genotyping PCRs used to confirm correct integration to the target gene. PCR primers should be designed to confirm integration at either 5′ UTR or 3′ UTR (or both), where the communal reverse primer (hsp70UTR_R) can be used for 5′ integration and the forward primer (PbU6prom_F) for 3′ integration. For 5′ integration, the forward primer should be designed upstream (UPS) of the HR1, and for 3’, the reverse primer should be designed downstream (DWS) of HR2. The UPS_F and DWS_R primers can then be combined to confirm the presence/absence of wild type (WT) locus, since the mutant parasites will generate a PCR product >7,000 bp, which is not amplified at the relevant PCR settings. Genomic DNA for the background (BG) line (in this case, Cas9-expressing parasites) should be run with each primer set as an additional control, to confirm bands are specific for integration. (B) Immunofluorescence assay used to confirm tagging of Rap2/3-3xMyc. Rabbit anti-Myc (target protein), rat anti-EXP1 (dense granule marker), and 4′,6-diamidino-2-phenylindole (DAPI). Scale bar = 5 μm. The parasite line from panel B was established using PbHiT in Jonsdottir et al. [26].

3. Genotyping PCRs to confirm integration (see Figure 3).

a. For each gene, design two primers to confirm integration at the 5′ and 3′ end of the gene by selecting a region upstream (UPS) of HR1 for 5′ integration (UPS_F) and a region downstream (DWS) of HR2 for 3′ integration (DWS_R).

b. The following communal primers (Table 1) can be paired with the gene-specific primers: hsp70UTR_R and PbU6prom_F, mentioned in step C4b. To check the 5′ integration, combine UPS_F with hsp70UTR_R, and to check integration at the 3′ end, combine PbU6prom_F with DWS_R.

c. To check the presence of wild-type parasites, pair the UPS_F and DWS_R primers. Since the entire vector is integrated if successful integration has occurred, this band would be around 7,000 bp and therefore will not be amplified using the PCR cycling conditions.

d. Always include genomic DNA from wild-type parasites as a control for each PCR combination.

e. When setting up the PCRs, use 10 ng of genomic DNA and then follow the generic protocol for either 2× GoTaq or 2× DreamTaq polymerases. For GoTaq, annealing temperature should be 2 °C below the calculated melting temperature, and for DreamTaq, 5 °C below the melting temperature. Under both conditions, the extension temperature should be 62 °C. Sometimes, optimisation is required, depending on the area of amplification.

*Note: We highly recommend confirming successful integration of tagging lines using immunofluorescence assays and western blotting.*

4. Removing the human dehydrofolate reductase drug cassette using negative selection with 5-fluorocytosine treatment.

a. If desired, the drug selection cassette can be removed from mutant parasites, making them non-resistant to pyrimethamine. The PbHIT vector includes duplicated PbDT3′UTR sequences flanking the selection cassette (see Figure 2Aii). These identical sequences allow homologous recombination–mediated excision of the entire marker cassette. When 5-fluorocytosine is added, parasites retaining the h*dhfr*/y*fcu* gene convert it into toxic 5-fluorouracil and are eliminated, while those that have excised the cassette survive. This is particularly useful if the plan is to transfect a different construct into the same parasite line.

b. After the parasites are confirmed to have the desired integration, infect a mouse from parasite stock and smear on days 3–4 to count parasitemia as described previously. Add one blood drop from a tail bleed and place it into a 1.5 mL tube containing 500 μL of RPMI.

c. Count cells using a Neubauer hemacytometer according to the protocol.

d. Clone the line by limiting dilution, based on cell counts and parasitemia, by inoculating 4–8 mice with a single parasite and provide pyrimethamine-treated water.

e. Monitor parasitemia using daily blood smears from day 4 post-infection. If parasites are visible in a blood smear, wait for the parasitemia to reach >0.1% and then move the mouse/mice to a new cage and provide drinking water with 5-fluorocytosine. Place foil around the bottle to protect it from light.

f. When parasitemia reaches 3%–5% post 5-fluorocytosine treatment, collect the blood via heart puncture as described in steps D2b–d.

g. Extract genomic DNA as before and confirm that 1) there is no wild-type genomic DNA and 2) there is no human dehydrofolate reductase drug cassette present, using either 2× GoTaq or 2× DreamTaq polymerase mix as described previously.

**Figure 4.**
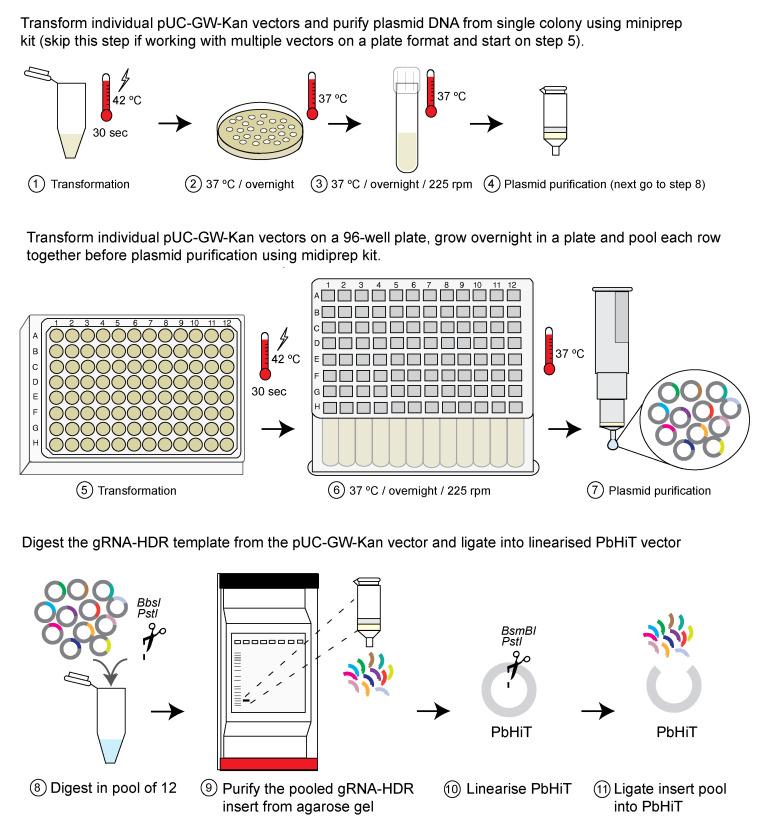
Scaling up the ligation step. The ligation step can be scaled up by pooling together 12× vectors, either from individual vector DNA (steps 1–4) or from pooled bacteria culture (steps 5–7). Figure adapted from Jonsdottir et al. [26].

**G. Scaling up the ligation step when generating vector pools (see Figure 4)**

1. Purify plasmid DNA from a single colony per plasmid as described in step B1 or grow a glycerol stock of pUC vectors in a 96-well plate as described in step H1.

**Pause point:** The protocol can be paused here, and DNA can be stored at -20 °C until used.

2. Digest pUC-GW-Kan vector in a pool of 12.

a. Pool together DNA from 12× pUC-GW-Kan vectors into a 1.5 mL tube to make a total of 2 μg of DNA.

b. Digest the vector pool using 1 μL of BbsI, 1 μL of PstI, 5 μL of SmartCut buffer, and water up to 50 μL.

c. Incubate the digest at 37 °C overnight.

d. Add 10 μL of 6× DNA loading dye to the digest, run the entire digest on a 1% agarose gel, and cut the pooled insert and gel extract as explained in detail in step B2 for a single vector.

**Pause point:** The protocol can be paused here, and DNA can be stored at -20 °C until used.

3. Ligate the pooled insert into the linearised PbHiT vector exactly as described in section C.

4. Transform 2 μL of the ligation reaction as described in steps B1c–d.

*Note: Leftover ligation reaction can be stored at -20 °C to use later if needed. Label the tube clearly.*

5. Spread all the cells on an LB agar plate containing ampicillin and grow overnight at 37 °C.

**Pause point:** The agar plate can be stored at 4 °C, and positive colonies can be used later. We recommend not storing it for longer than 2 weeks, as there is a risk of contamination while storing the plate.

6. Either set up individual bacteria cultures with single colonies as described in step B1f if a stock of single vectors is required, or pool together all the colonies using a plate spreader and grow in 500 mL of LB with ampicillin if pooled vectors are desired. If using a vector pool, DNA needs to be purified using a Plasmid Midi Kit according to the kit protocol, instead of miniprep.

**Pause point:** The protocol can be paused here, and DNA can be stored at -20 °C until used.

7. Confirm successful ligation using Sanger sequencing (individual vectors) or NGS (pooled vectors).

**H. Pooled vector transfections and time-course sampling (see Graphical overview)**

1. Prepare the vector pool for transfection.

a. Grow individual pUC-GW-Kan vectors (from glycerol stocks prepared in step B1) in 1 mL of LB broth supplemented with kanamycin (50 μg/mL) in a 96 deep-well plate. Incubate overnight at 37 °C with shaking at 225 rpm.

b. The following day, pool cultures in groups of 12 (e.g., each row = one pool), purify plasmids, and perform pooled ligations into the PbHiT vector as described in section G. Purify the final ligated vectors using a Plasmid Midi kit.

**Pause point:** The protocol can be paused here, and DNA can be stored at -20 °C until used.

c. To generate transfection pools, combine approximately 450 ng of each PbHiT vector. For a pool of 12 vectors, this corresponds to ~5,400 ng in total. Pools can be scaled up to include as many as 96 vectors. Optionally, spike in control vectors at 450 ng per vector.

*Note: We recommend including a selection of control vectors for any high-throughput knockout/knockdown screen [9]. For general asexual blood stage growth, for* P. berghei, *we recommend using well-characterised genes, such as* PBANKA_0515000 (p25), PBANKA_0933700 (mapk2), PBANKA_1013300 (mapk1*), and* PBANKA_1037800 (soap*) as dispensable controls and* PBANKA_0211000 (tim50), PBANKA_0706400 *(conserved)*, PBANKA_1039700 (Apicox19*), and* PBANKA_1214100 (tubulin binding cofactor c*) as essential controls. If interested in a specific phenotype/process or a different stage, we recommend using at least four genes (two essential and two dispensable) that have been well characterised and can serve as internal controls for your assay.*

d. Linearise the pool overnight with AvrII by adding 0.5 μL of enzyme per microgram of pooled vectors (e.g., 10.8 μL of AvrII for a 12-vector pool), together with 30 μL of SmartCut buffer and water to a final volume of 300 μL. Proceed as described in step C5. Resuspend the vector in 30 μL of Milli-Q water and run 1 μL on a 0.7% agarose gel to confirm the presence of the linearised plasmid prior to transfection.

**Pause point:** The protocol can be paused here, and DNA can be stored at 4 °C until used. If storing the DNA, run the DNA on a gel after storage to confirm it is still of good quality before transfection.

2. Transfect the PbHiT-gRNA-HDR linearised vector into Cas9-expressing schizonts.

a. Infect a mouse with Cas9-expressing parasite from 200 μL of frozen parasite stock as previously described.

b. On days 3–4 post-infection, confirm if infection was successful and infect a rat with the mouse blood.

*Note: It is recommended to use rats when conducting screens that require multiple pools of vectors.*

c. Once the rat is infected, collect blood as described previously and set up a 200 mL schizont culture as indicated for mice in section D.

d. Transfect the linearised vector into Cas9-expressing schizonts following the procedure in section E. Here, divide the DNA/schizont/P3 samples between 3× cuvettes of the 16× Nucleocuvette strip. After electroporation, collect all samples into a 1.5 mL tube, resuspend in 300 μL of RPMI (room temperature), and divide between 3× insulin syringes.

e. Inject transfected schizonts into three mice as previously described.

f. Provide mice with pyrimethamine-treated water the day after transfection.

3. Time-course sampling.

a. Immediately after transfection, collect an input sample by washing the three electroporation cuvettes with 200 μL of PBS.

**Critical:** It is important to have the sample input for the pooled transfection to know exactly the vector composition and the amount of each vector at the start.

b. Boil sample at 95 °C for 5 min, centrifuge at 16,200× *g*, and store supernatant at -20 °C.

c. From day 4 to day 8 post-transfection, collect 20 μL of blood by tail bleed into a tip preloaded with a small amount of heparin. To do this, the tip of the tail needs to be cut off first.

*Note: Start monitoring parasitemia from day 3 post-transfection. Only start blood collection when you start seeing parasites.*

d. Transfer the blood into 200 μL of PBS containing 10 μL of heparin.

**Critical:** Be careful to collect blood into the tip relatively fast to avoid coagulation of the blood during collection.

e. Keep all samples at 4 °C until the final collection point.

**I. Preparation of samples for NGS and analysis**

1. Extract genomic DNA from all samples.

a. Resuspend the blood pellet in 180 μL 1× PBS and extract genomic DNA as described in step F2. Perform double elution in ultra-pure water.

b. Store DNA at -20 °C until use.

2. Set up a PCR master mix to amplify the barcode region (across the gRNA area).

a. Transfer all PCR reagents to a PCR-specific hood [dNTP mix, Advantage Taq polymerase (keep on ice), Advantage Taq buffer, ultrapure water, PAGE purified primers, and PCR plate].

*Note: All reagents should only be opened inside a PCR-specific hood to avoid any contamination.*

b. Use PAGE-purified primers to amplify the barcode region, BC_pHiT_illumina_F and BC_pHiT_illumina_R (Table 1).

c. Always include a water control in the last well of the PCR plate.

d. In a tube, prepare enough master mix to have 25 μL of final PCR reaction per sample: 0.5 μL of polymerase, 0.5 μL of forward primer, 0.5 μL of reverse primer, 2.5 μL of buffer, 0.5 μL of dNTPs, 15.5 μL of water, and 5 μL of genomic DNA (not included in master mix).

*Note: Add the polymerase last, keep on ice until use, and then place immediately back on ice while finishing setting up the PCR.*

e. Carefully pipette 20 μL of master mix into each well on a PCR plate.

*Note: If you have a lot of samples, it can be useful to use a reagent reservoir and multi-pipette into the wells.*

f. Add 5 μL of ultrapure water into the last well (water control) and resuspend.

g. Add 5 μL of genomic DNA into relevant wells and resuspend.

h. Place an aluminium film and seal using a roller.

i. Place the plate into a PCR machine that fits a 96-well plate using these settings: 95 °C denaturation for 5 min, 35 cycles of 95 °C (30 s), 55 °C (20 s), and 68 °C (8 s), and then a final extension at 68 °C for 10 min.

*Note: If using different primers, PCR settings might need adjusting.*

j. Run 1 μL of the PCR reaction on a 1% agarose gel to confirm amplification of 250 bp.

*Note: Some samples might not yield any product or be less visible and might need adjusting the DNA volume. If this happens, carefully note which wells worked and which ones you need to repeat, as you need to use the DNA from PCR1 in the next step.*

**Pause point:** The protocol can be paused here, and the plate with PCR1 can be stored at 4 °C.

3. Set up a nested PCR to add Illumina adaptor sequences and index primers to enable sample multiplexing.

a. Set up the PCR master mix as described in the previous step inside a PCR cabinet.

b. Use a generic PAGE purified PE 1.0 primer and pair with PAGE purified index primers (1 per sample); sequences can be found in Bushell et al. [9].

*Note: When handling several samples, it is recommended to prepare the index primers separately on a 96-well PCR plate in order to facilitate multi-pipetting for PCR2.*

c. In a tube, set up a 50 μL PCR master mix as before, except this time, only the generic PE 1.0 primer is included in the master mix.

d. Pipette 44 μL of PCR master mix into each well of a 96-well plate (this time, include two wells for water control).

*Note: If you have a lot of samples, it can be useful to use a reagent reservoir and multi-pipette into the wells.*

e. Pipette 1 μL of index primers into relevant wells.

f. Add 5 μL of PCR1 reaction into relevant wells (do the same for the old water control well).

g. Add 5 μL of water to the new water control well.

h. Place an aluminium film and seal using a roller.

i. Place the plate into a PCR machine that fits a 96-well plate using these settings: 95 °C denaturation for 2 min, 10 cycles of 95 °C (30 s) and 68 °C (15 s), followed by a final extension at 68 °C for 5 min.

*Note: If using different primers, PCR settings might need adjusting.*

j. Run 1 μL of the PCR reaction on a 1% agarose gel to confirm amplification.

*Note: Some samples might not yield any product or be less visible and might need adjusting the DNA volume. If this happens, carefully note which wells worked and which ones you need to repeat, as you need to purify the final DNA.*

**Pause point:** The protocol can be paused here, and the plate with PCR2 can be stored at 4 °C.

4. DNA purification and pooling for NGS analysis.

a. Once all the PCR2 reactions have been confirmed by agarose gel, purify the DNA using the MinElute 96 UF PCR Purification kit following the purification procedure.

b. Transfer PCR2 samples to the 96-well plate from the kit and use vacuum for 10 min (until wells are dry).

*Note: Tape over any unused wells and hold the plate down to ensure a proper seal for the vacuum (need 800 mbar).*

c. Add 50 μL of ultra-pure water into each well and use the vacuum for 10 min. Tap the plate down on tissue and then for 4 min until wells are dry.

d. Add 30 μL of ultra-pure water to each well and incubate for 5 min at room temperature.

e. Then, use a multi-channel pipette to resuspend the liquid up and down around 10 times and then transfer the elution to a clean 96-well PCR plate and seal with a roller and an aluminium cover.

**Pause point:** The protocol can be paused here, and the plate with purified DNA can be stored at 4 °C.

f. Use the Qubit assay kit to measure the DNA concentration for each sample according to the kit protocol.

g. Prepare a DNA library pool by pooling together 125 ng of each sample.

*Note: If using an Illumina sequencing service platform, they can perform additional quality controls and qPCR before running the samples.*

h. Send the DNA library pool for Illumina sequencing, where samples are diluted to 1.5 pM and then loaded as a low cluster density (4 × 10^5^ clusters/mm^2^) for MiSeq.

*Note: If using the Illumina NextSeq sequencing platform, perform additional purification using SPRIselect beads (1:0.8 library:beads ratio) to remove adapter dimers. Dilute to 0.5 pM and load at a cluster density of 7 × 10^4^ clusters/mm^2^.*

**J. Growth rate statistical analysis for CRISPR screens**

Calculate growth rates from pooled vector transfection time-course samples. This can be done using suitable code in R using a custom R pipeline. We provide code and examples of its use at https://github.com/henriksson-lab/pbhit_analyzer. In summary, the code describes the following analysis steps:

1. Extract gRNA abundances from each sequencing sample by identifying the U6 promoter sequence (CAATATTATT) and the following gRNA sequence. Only gRNAs that exactly match the expected reference list should be retained.

2. For each sample (mouse and timepoint), calculate gRNA abundance as a fraction of total counts. Normalise relative abundances by dividing by the sum of counts from control gRNAs targeting known dispensable genes.

3. Fit a logistic growth function to the relative abundance data. To ensure robust fits, fix the carrying capacity at 1, while allowing the relative growth rate (RGR) and initial abundance to be estimated. Fits can be performed using the nonlinear least squares solver in the R package minpack.lm (v1.2–4).

4. If a fit fails to converge (e.g., when coverage is low or variance is high), exclude the data point from further analysis. This functions as an outlier removal step.

5. For each gene, calculate the growth rate as the mean of its corresponding gRNAs. Propagate variances assuming independent variables.

6. Visualise results in RShiny, ggplot2, or plotly, and assess classification performance using receiver operating characteristic (ROC) curves with the plotROC package.

7. All scripts used to extract gRNA counts and perform growth rate analysis are available at https://github.com/henriksson-lab/pbhit_analyzer.

## Data analysis

Growth rate derived from time course samples from pooled vector screens can be analysed as described in section J, according to previously published analysis [26]. Scripts used to extract counts from sequencing data can be found on GitHub (https://github.com/henriksson-lab/pbhit_analyzer). These scripts are derived from our previously published code but were generalised to enable other researchers to analyse their own data.

## Validation of protocol

This protocol (or parts of it) has been used and validated in the following research article(s):

• Jonsdottir et al. [26]. A scalable CRISPR-Cas9 gene editing system facilitates CRISPR screens in the malaria parasite *Plasmodium berghei*. Nucleic Acids Research (Figures 3–5 and Supplemental Figures S3 and S5–S11).

## General notes and troubleshooting

**General notes**

1. We recommend always testing at least two gRNAs per gene targeted.

2. This protocol has the possibility of being adapted to other apicomplexan parasites that rely on homologous recombination. However, the length of the homology region has been optimised specifically for *P. berghei*, and longer homology arms might be required for optimal integration when applied to other organisms.

3. The gRNA-HDR fragment can be made using PCRs instead of ordering a synthetic fragment in a vector, especially when only targeting a few genes.

**Troubleshooting**

**Table:** 

Problem	Possible reason	Solution
**gRNA-HDR fragment cloning**		
Very few or no colonies after transformation of pUC-GW-Kan.	Competent cells not efficient; heat shock timing/temperature incorrect; recovery too short; LB not prewarmed.	Use fresh high-efficiency competent cells; confirm 30 s at 42 °C; allow ≥45 min recovery in warm LB; prewarm agar plates.
Miniprep yields low DNA.	Small culture volume or poor growth.	Increase culture volume (up to 10 mL); incubate for 14–16 h shaking at 225 rpm.
Restriction digest with BbsI/PstI incomplete.	Enzyme activity reduced; DNA too concentrated.	Use fresh enzymes; lower DNA input or extend incubation.
Gel extraction yields little DNA.	Too much agarose excised; elution volume too high.	Excise the smallest possible band; elute in ≤15 μL warm water; re-elute through the column.
**PbHiT vector preparation**		
PbHiT vector digestion with BsmBI unsuccessful (unspecific bands, smear).	Star activity.	Avoid over-digestion; quantify DNA before digest.
Ligation yields no colonies.	Incorrect insert:vector ratio; ligase inactive; degraded insert.	Recalculate with NEBio Calculator; use fresh ligase and buffer; confirm insert integrity on gel.
Colony PCR gives no band or wrong size.	Low template.	Leave the tip on the PCR mix for a longer time; increase the 95 °C step to 10 min to increase bacterial lysis and DNA extraction.
**Schizont culture**		
Few or no schizonts after overnight culture.	Insufficient parasitemia; culture conditions suboptimal.	Bleed mice with higher parasitemia; ensure correct gassing and no leakage.
**Transfection**		
Low transfection efficiency.	Schizonts not mature; DNA not fully linearised; electroporation settings incorrect.	Collect late (22–24 h) schizonts; confirm linearisation by gel; check electroporation program.
No parasites detectable in mice post-injection.	Poor schizont quality; DNA degraded; electroporation failed; poor injection technique.	Reassess schizont prep; verify DNA integrity; repeat with new electroporation cuvette.
**Genotyping**		
No PCR product from genomic DNA.	High or low DNA concentration.	Verify DNA quality and quantity; adjust template input; perform PCR to check extraction.
Nonspecific PCR bands.	Annealing temperature too low.	Optimise annealing temperature.
**Large-scale pooled ligation**		
Low yield after pooled ligation.	Insert:vector ratio incorrect; ligase inactive.	Optimise ratio; use fresh ligase/buffer; confirm insert integrity.
**Pooled transfections and sampling**		
No parasites expand after pooled transfection.	Low vector quality; AvrII digest incomplete; schizonts unhealthy.	Re-purify plasmids with Midi Kit; confirm digest on gel; ensure schizont culture quality.
Blood samples clotted during collection.	Not enough heparin.	Ensure pipette tip contains heparin; mix immediately after collection; collect samples quickly.
**Sequencing library prep**		
Uneven sequencing coverage across gRNAs.	Primer bias; low DNA input.	Optimise primer design; increase DNA input; balance multiplexing carefully.
**Analysis**		
Growth curve fitting does not converge.	Lack of reads for the given vector; model is unsuitable for the given context.	Manually inspect the relative abundances. If the counts are low, sequence deeper. Variance of relative abundance is higher at lower counts.
